# N-Acetyltransferase 9 Inhibits Porcine Reproductive and Respiratory Syndrome Virus Proliferation by N-Terminal Acetylation of the Structural Protein GP5

**DOI:** 10.1128/spectrum.02442-22

**Published:** 2023-01-25

**Authors:** Xiaoyang Li, Ruiqi Sun, Yanyu Guo, Huixia Zhang, Ruyu Xie, Xubin Fu, Lei Zhang, Lilin Zhang, Zexing Li, Jinhai Huang

**Affiliations:** a School of Life Sciences, Tianjin University, Tianjin, China; b Tianjin Ringpu Bio-technology Co., Ltd., Tianjin, China; Shandong First Medical University

**Keywords:** Nat9, porcine reproductive and respiratory syndrome virus, N-terminal acetylation, GP5, transcription factors

## Abstract

Porcine reproductive and respiratory syndrome virus (PRRSV) is a serious threat to the global swine industry. As a typical immunosuppressive virus, PRRSV has developed a variety of complex mechanisms to escape the host innate immunity. In this study, we uncovered a novel immune escape mechanism of PRRSV infection. Here, we demonstrate for the first time that the endoplasmic reticulum (ER)-resident N-acetyltransferase Nat9 is an important host restriction factor for PRRSV infection. Nat9 inhibited PRRSV proliferation in an acetyltransferase activity-dependent manner. Mechanistically, glycoprotein 5 (GP5) of PRRSV was identified as interacting with Nat9 and being N-terminally acetylated by it, which generates a GP5 degradation signal, promoting the K27-linked-ubiquitination degradation of GP5 to decrease virion assembly. Meanwhile, the expression of Nat9 was inhibited during PRRSV infection. In detail, two transcription factors, ETV5 and SP1, were screened out as the key transcription factors binding to the core promoter region of Nat9, and the PRRSV nonstructural protein 1β (Nsp1β), Nsp4, Nsp9, and nucleocapsid (N) proteins were found to interfere significantly with the expression of ETV5 and SP1, thereby regulating the transcription activity of Nat9 and inhibiting the expression of Nat9. The findings suggest that PRRSV decreases the N-terminal acetylation of GP5 to support virion assembly by inhibiting the expression of Nat9. Taken together, our findings showed that PRRSV has developed complex mechanisms to inhibit Nat9 expression and trigger virion assembly.

**IMPORTANCE** To ensure efficient replication, a virus must hijack or regulate multiple host factors for its own benefit. Understanding virus-host interactions and the molecular mechanisms of host resistance to PRRSV infection is necessary to develop effective strategies to control PRRSV. The N-acetyltransferase Nat9 plays important roles during virus infection. Here, we demonstrate that Nat9 exhibits an antiviral effect on PRRSV proliferation. The GP5 protein of PRRSV is targeted specifically by Nat9, which mediates GP5 N-terminal acetylation and degradation via a ubiquitination-dependent proteasomal pathway. However, PRRSV manipulates the transcription factors ETV5 and SP1 to inhibit the expression of Nat9 and promote virion assembly. Thus, we report a novel function of Nat9 in PRRSV infection and elucidate a new mechanism by which PRRSV can escape the host innate immunity, which may provide novel insights for the development of antiviral drugs.

## INTRODUCTION

Porcine reproductive and respiratory syndrome virus (PRRSV) is one of the most devastating viruses threatening the swine industry worldwide (1). PRRSV is a single-stranded, positive-sense RNA virus with an approximately 15-kb genome (2). There are 11 open reading frames (ORFs) in the PRRSV genome, ORF1a, ORF1b, ORF2a, ORF2b, ORF3 to -7, ORF5a, and ORF2 transframe (TF), which collaborate to encode 9 structural proteins and 16 nonstructural proteins (Nsps) of PRRSV with a complex mechanism (3, 4). The matrix (M) protein and glycoprotein 5 (GP5) are the major components of the virus capsule, and they are important for the attachment and internalization of PRRSV through clathrin-mediated endocytosis (5, 6). Nonstructural proteins and structural proteins of PRRSV play important roles in modulating host antiviral responses to favor PRRSV’s evasion and proliferation (7–10). For example, Nsp1α, Nsp1β, Nsp2, Nsp4, Nsp11, and the nucleoprotein (N) have been suggested to suppress interferon (IFN) production to disarm the innate immunity of the host through various mechanisms (11, 12). Nsp4 and Nsp10 were indicated as apoptotic proteins that induce apoptosis in cells and in pigs after PRRSV infection (13). N and Nsp2 have been reported to be activators of NF-κB activation, whereas Nsp1α, Nsp1β, Nsp2, Nsp4, and Nsp11 are known as suppressors of NF-κB activation (14). Nsp11 inhibits interleukin-1β (IL-1β) production and the NLRP3 inflammasome in microglia that is dependent on its endoribonuclease activity (15, 16). Nsp1β and Nsp11 are reported to inhibit tumor necrosis factor alpha (TNF-α) production induced by highly pathogenic PRRSV and the differential TNF-α production in porcine alveolar macrophages (PAMs) (17, 18).

It is important to identify new host defense line and restriction factors besides the classical antiviral responses mentioned above, which will help us better understand the molecular and cellular mechanisms for PRRSV’s pathogenesis. N-terminal acetylation is one of the most prevalent co- and posttranslational modifications in eukaryotic cells, and it is catalyzed by the N-acetyltransferase (NAT) family members, which employ distinctive amino acid features for substrate specificity recognition. Recent studies have indicated that NATs play important roles during virus infection. For example, NatB-mediated N-terminal acetylation of PA-X is required for the shutoff activity of PA-X and viral polymerase activity of influenza virus (19). NAA60 promotes influenza A virus infection in a cell type- and influenza A virus (IAV) strain-independent manner (20). Nat8 promotes enterovirus 71 replication by stabilizing the viral 2B, 3AB, and 3C proteins (21). However, whether NATs are involved in PRRSV infection and proliferation has not been studied.

In this study, we focused on the transcriptional changes of NAT members in 3D4/21 cells after PRRSV infection, and we identified Nat9 as a host restriction factor for PRRSV infection. Molecular mechanism studies indicate that Nat9-mediated N-terminal acetylation promotes GP5 proteolysis through K27-linked ubiquitination. This N-terminal acetylation modification is responsible for most PRRSV GP5 protein, indicating that N-terminal acetylation is a novel line of host defense against PRRSV infection. Furthermore, Nsp1β, Nsp4, Nsp9, and N inhibit the transcriptional level of Nat9 to antagonize its restriction, which provides new insights into the pathogenesis and immune suppression of PRRSV infection.

## RESULTS

### Nat9 expression is downregulated after PRRSV infection.

**FIG 1:**
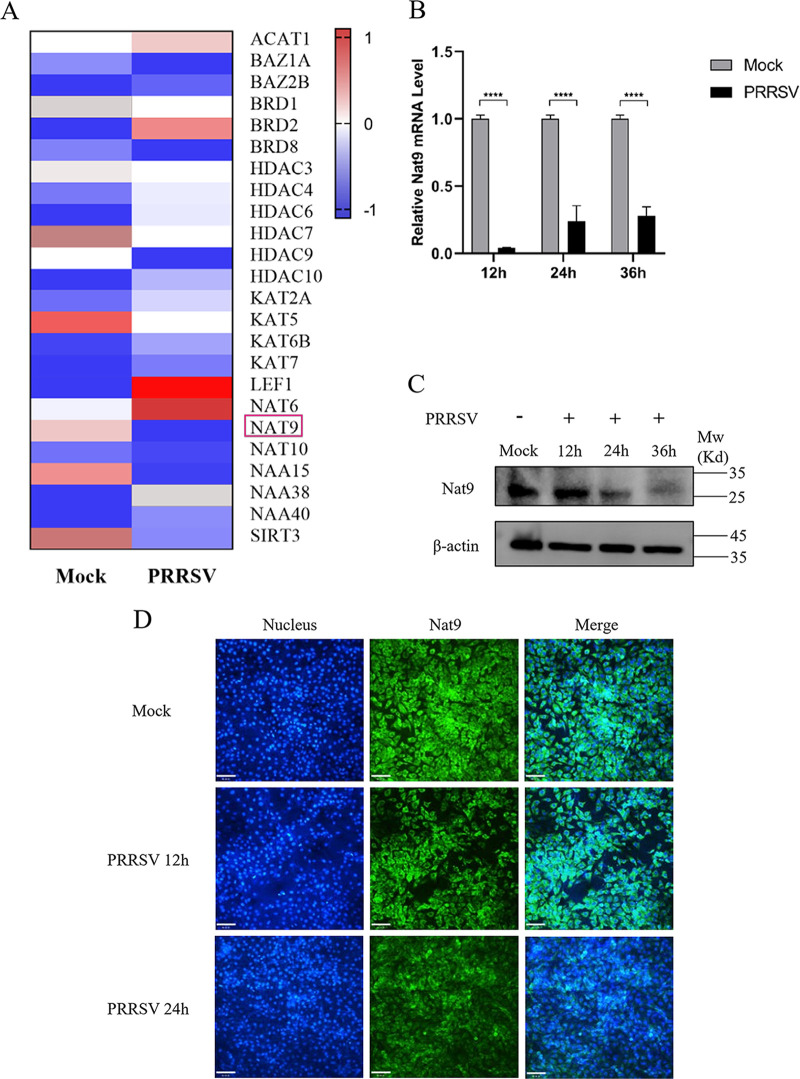
Porcine Nat9 is downregulated in 3D4/21 cells by PRRSV. (A) The heatmap of acetylation-related differentially expressed genes was generated using GraphPad Prism 8. Red indicates an upregulation in expression of at least 1-fold. (B) qRT-PCR analysis of Nat9 in 3D4/21 cells inoculated without or with 0.5 MOI PRRSV at the indicated times. (C) 3D4/21 cells were either mock infected or infected with PRRSV at 0.5 MOI for 12 h, 24 h and 36 h. The cells were then harvested to detect Nat9 by Western blotting using a mouse anti-Nat9 polyclonal antibody. (D) 3D4/21 cells were either mock infected or infected with PRRSV at 0.5 MOI for 12 h and 24 h. The cells were fixed and stained with a mouse anti-Nat9 antibody, followed by FITC-conjugated anti-mouse IgG (green). Nuclei were stained with DAPI (blue). Cells were observed under a laser confocal imaging analysis system. Scale bar = 7 μm. Error bars show SD. *, *P* < 0.05; **, *P* < 0.01; ***, *P* < 0.001; ****, *P* < 0.0001 (one-way ANOVA followed by Bonferroni posttest). Data are representative of three independent experiments.

To determine whether N-terminal acetylation modification was involved in PRRSV infection and proliferation, we first focused on the transcriptional changes of NAT members in 3D4/21 cells after PRRSV infection (22, 23). A total of 24 acetylation-related genes were altered after PRRSV infection, including lysine acetyltransferase, lysine deacetylase, bromodomain-containing protein, and N-acetyltransferase genes (Table S1 in the supplemental material). Little research on the functions of Nat9 has been reported, and reports of Nat9 impacting virus proliferation are largely unknown. So Nat9 was selected for further study (Fig. 1A). To confirm our transcriptome analysis results, 3D4/21 cells were infected with PRRSV for various times and the dynamic mRNA and protein levels of Nat9 were detected by quantitative reverse transcription-PCR (qRT-PCR) and Western blotting. As shown by the results in Fig. 1B and C, both the mRNA and protein levels of Nat9 were decreased after PRRSV infection. Furthermore, immunofluorescence experiments showed that endogenous Nat9 was gradually decreased after PRRSV infection (Fig. 1D). All results indicate that Nat9 is associated with PRRSV infection.

### Nat9 inhibits PRRSV proliferation in 3D4/21 cells.

**FIG 2:**
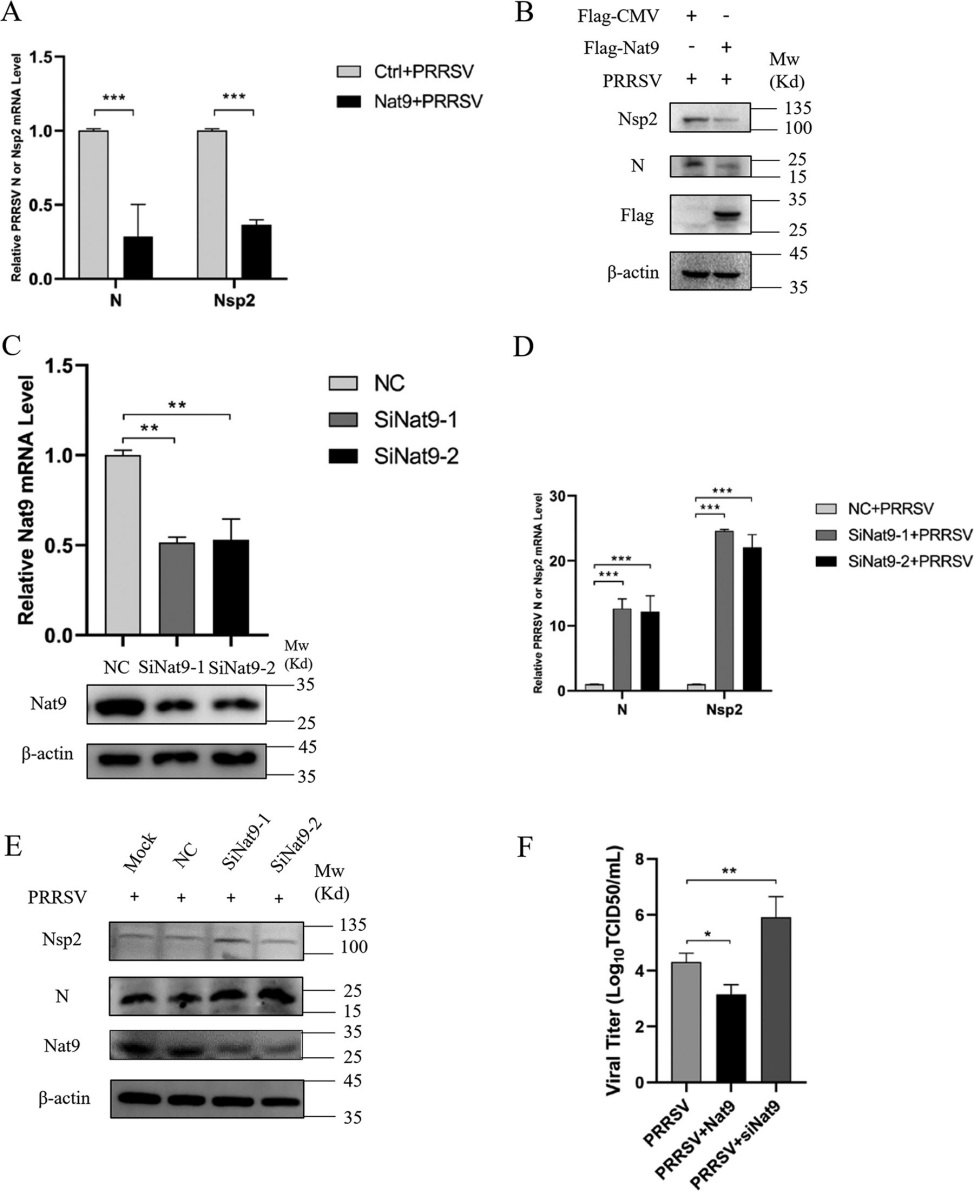
Porcine Nat9 inhibited viral replication of PRRSV. Flag-tagged Nat9 plasmid, negative control (NC), siRNA1 (siNat9-1), and siRNA2 (siNat9-2) targeting Nat9 were transfected into 3D4/21 cells at the indicated times, after which the cells were infected with 0.5 MOI PRRSV and then collected at 12 h or 24 h postinfection. (A, D) PRRSV N and Nsp2 mRNA was detected by qRT-PCR. (C) siRNA interference effects were detected by anti-Nat9 PAb immunoblotting and qRT-PCR at 24 h posttransfection. (B, E) The expression of PRRSV N and Nsp2 was shown by Western blot analysis. 3D4/21 cells were transfected with Flag-Nat9 or siRNA and then infected with PRRSV JXwn06 at an MOI of 1. At 24 h postinfection, PRRSV titers in the supernatants were measured by plaque assay in 3D4/21 cells (F). Error bars show SD. *, *P* < 0.05; **, *P* < 0.01; ***, *P* < 0.001 (analysis of one-way ANOVA followed by Bonferroni posttest). Data are representative of three independent experiments.

To test whether Nat9 influenced PRRSV proliferation, Nat9 was overexpressed in 3D4/21 cells and both the mRNA and protein levels of N and Nsp2, which were directly related to PRRSV replication, were detected. We found that overexpression of Nat9 significantly downregulated both the mRNA and protein levels of N and Nsp2 (Fig. 2A and B). To further confirm the function of endogenous Nat9, we synthesized two pairs of small interfering RNAs (siRNAs) targeting Nat9 and tested the knockdown efficiency in 3D4/21 cells (Fig. 2C). We found that both the mRNA and protein levels of N and Nsp2 were increased after knockdown of Nat9 (Fig. 2D and E). Consistent with this, overexpression of Nat9 significantly decreased the PRRSV titer compared to the results for the empty vector control (Fig. 2F). These data indicate that the changes in cellular Nat9 are associated with PRRSV proliferation and that Nat9 is a host restriction factor for PRRSV infection.

### Nat9 inhibits PRRSV proliferation dependent on its N-acetyltransferase activity.

**FIG 3:**
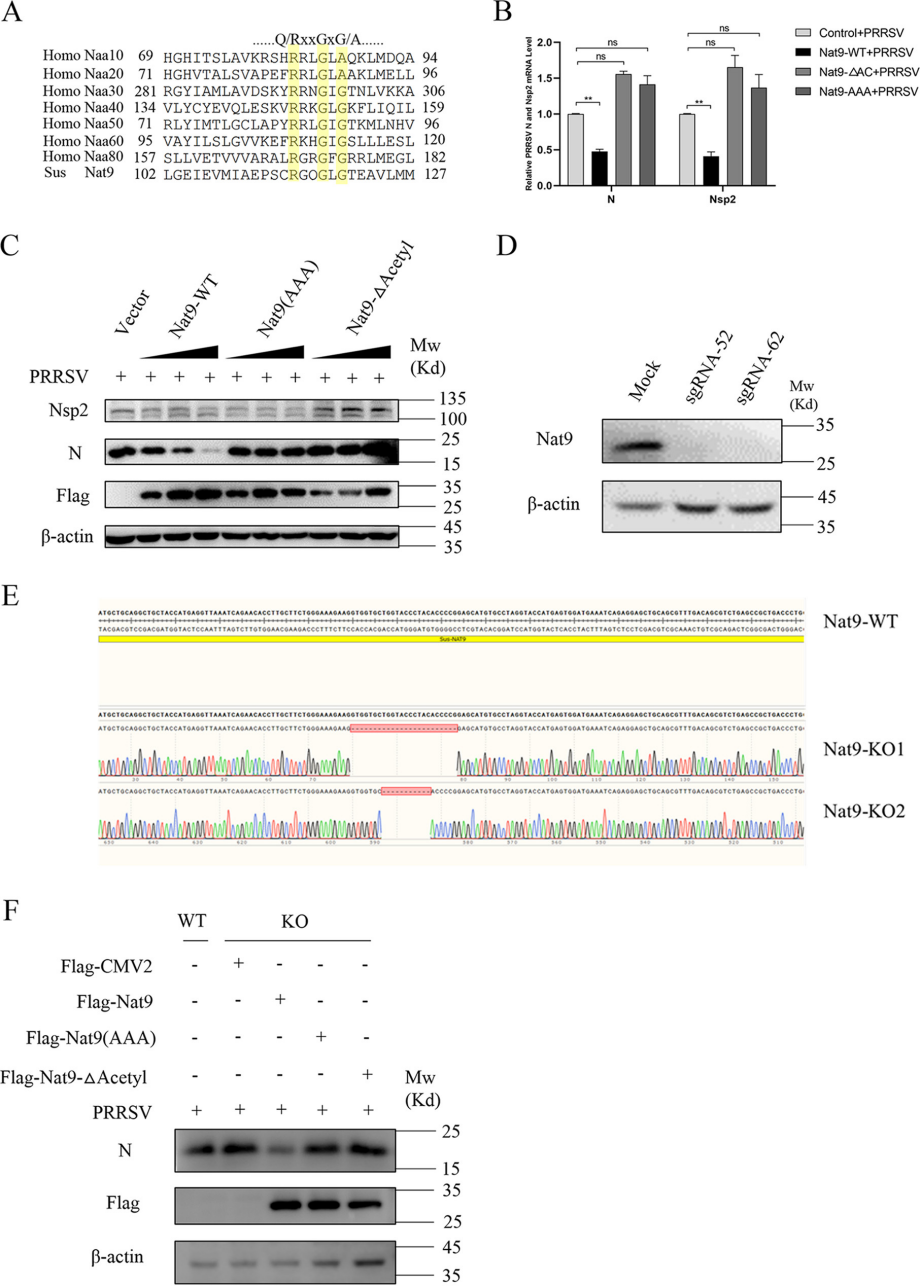
Porcine Nat9 inhibits PRRSV proliferation through its N-acetyltransferase activity. (A) Nat9 has the acetyl-CoA binding motif conserved in all known NATs. (B) Flag-tagged Nat9-WT or mutant plasmids were transfected into 3D4/21 cells, after which the cells were infected with 0.5 MOI PRRSV and collected 24 h postinfection. PRRSV N and Nsp2 mRNA was detected by qRT-PCR. (C) Flag-tagged Nat9-WT or mutant plasmids were transfected into 3D4/21 cells at specified doses (0.5 μg, 1 μg, and 1.5 μg), after which the cells were infected with 0.5 MOI PRRSV and then collected 24 h postinfection. The expression of PRRSV N and Nsp2 was detected by Western blotting. (D) Immunoblot analysis of Nat9 WT and KO 3D4/21 cells using anti-Nat9 antibody. (E) Sequence alignment results of the knockout cell line Nat9 gene and wild-type Nat9 gene. (F) Flag-tagged Nat9-WT or mutant plasmids were transfected into Nat9-KO cells, after which the cells were infected with 0.5 MOI PRRSV and then collected 24 h postinfection. PRRSV N was detected by Western blotting. Error bars show SD. **, *P* < 0.01; ns, not significant (analysis of one-way ANOVA followed by Bonferroni posttest). Data are representative of three independent experiments.

As Nat9 is predicted to be an N-acetyltransferase and the conserved acetyl-CoA binding motif, Q/RXXGXG/A (X can be any amino acid), is essential for acetyltransferase activity (24), we wanted to know whether the N-acetyltransferase activity was essential for the inhibition function of Nat9 on PRRSV proliferation (Fig. 3A). We deployed two different N-acetyltransferase-inactive mutants. Nat9-AAA (AXXAXA) had three alanine substitutions for R or G in the RXXGXG motif, and Nat9-ΔAc (ΔRGKGFG) had a deletion of all six amino acids in the RXXGXG motif. We next checked the influence of Nat9 wild type (Nat9-WT), Nat9-AAA, and Nat9-ΔAc on PRRSV proliferation. The mRNA levels of the N and Nsp2 genes were not decreased with Nat9-AAA or Nat9-ΔAc compared with their levels with Nat9-WT (Fig. 3B). Consistent with this, the protein levels of N and Nsp2 were decreased with Nat9-WT overexpression in a dose-dependent manner, whereas overexpression of Nat9-AAA or Nat9-ΔAc did not influence the protein levels of N and Nsp2 (Fig. 3C).

To substantiate our findings, we generated Nat9 knockout (KO) 3D4/21 cells using the clustered regularly interspaced short palindromic repeat (CRISPR)/Cas9 system (Fig. 3D and E), and the N protein of PRRSV was increased in the KO cells compared with that in the WT cells (Fig. 3F). To confirm that the phenomenon of KO cells was due to the endogenous Nat9 protein, we reconstituted the KO cells with Nat9-WT or the N-acetyltransferase-inactive mutants. As shown by the results in Fig. 3D, Nat9-WT could decrease the N protein level of PRRSV in the KO cells, whereas neither Nat9-AAA nor Nat9-ΔAc could rescue the phenomenon of KO cells (Fig. 3F). Taken together, these findings suggest that Nat9 inhibits PRRSV proliferation dependent on its N-acetyltransferase activity and that the RXXGXG motif is necessary for its acetyltransferase activity.

### Nat9 interacts with GP5 of PRRSV and promotes N-terminal acetylation of GP5.

The first and second N-terminal amino acid residues of the nascent protein are important for N-acetyltransferase recognition (25). A recent study suggests that Met-Leu (M-L) and Met-Arg (M-R) are important characteristic residues of α-tubulin and β-tubulin for recognition by *Drosophila* Nat9 (26). Based on this consideration and the finding that N-acetyltransferase activity is necessary for Nat9 to inhibit PRRSV proliferation, we focused on the N-terminal amino acids of structural and nonstructural proteins of PRRSV. Among these proteins, only GP5 contains Met-Leu (M-L) as the first two N-terminal residues in PRRSV strain JXwn06 (Fig. S1A), which was used for the experiments described above. Moreover, we searched and aligned the GP5 amino acid sequences of other PRRSV strains in NCBI and found that the N terminus of GP5 was not highly conserved but that Met-Leu (M-L), Met-Arg (M-R), and Met-Lys (M-K) were the most popular first two residues in different PRRSV strains (Fig. S2B). These findings encouraged us to investigate whether GP5 could be a substrate of Nat9 and modified with N-terminal acetylation by Nat9.

**FIG 4:**
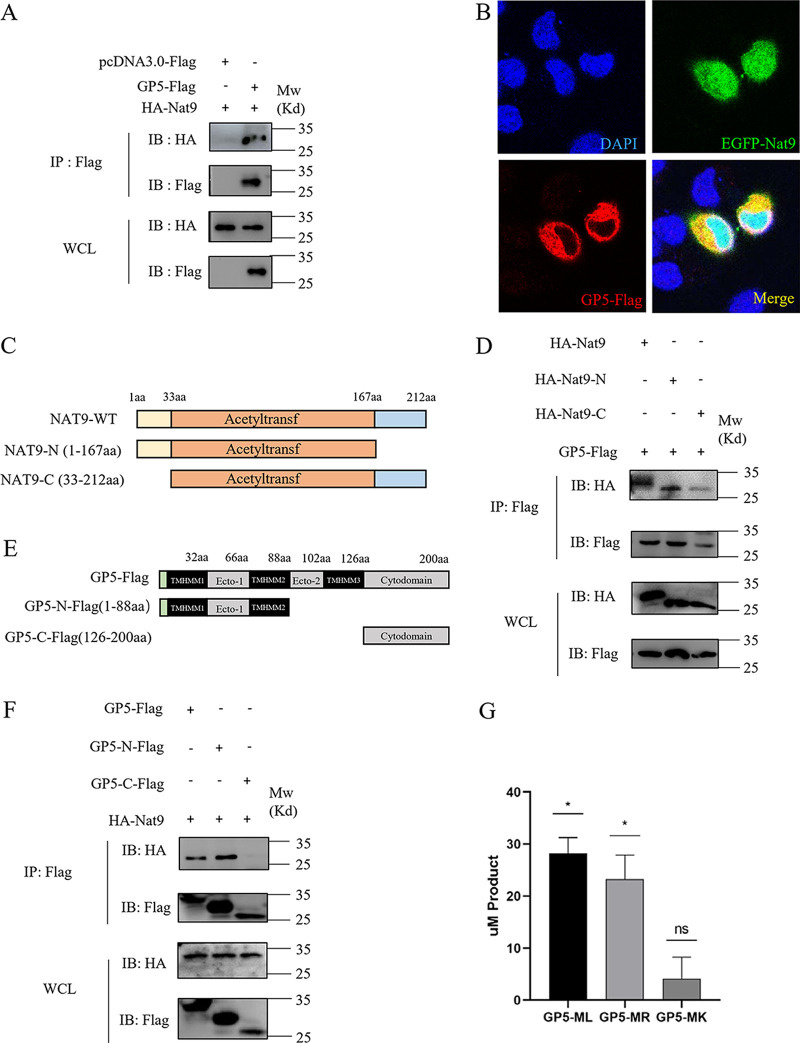
Porcine Nat9 interacts with the GP5 of PRRSV and acetylates the N terminus of GP5. (A) HEK293 cells were cotransfected with GP5-Flag and HA-Nat9 or vector plasmid. The cell lysates were then immunoprecipitated with an anti-Flag MAb and detected by Western blotting at 24 h posttransfection. (B) Cotransfection of EGFP-Nat9 with GP5-Flag into HeLa cells. The cells were fixed and stained with a mouse anti-Flag antibody, followed by Alexa Fluor 555 anti-mouse IgG (red). Nuclei were stained with DAPI (blue). Cells were observed under a laser confocal imaging analysis system. Scale bar = 14 μm. (C) Mapping of wild-type (WT) Nat9 and different key enzyme truncations of Nat9. The porcine Nat9 isoform consists of 212 amino acids with a single domain named the N-acetyltransferase domain. (D) HEK293T cells were cotransfected with GP5-Flag and different HA-tagged Nat9 deletion mutant expression plasmids. The cell lysates were then immunoprecipitated with an anti-Flag MAb and detected by Western blotting at 24 h posttransfection. (E) Schematic diagram of different fragments of GP5. The predicted topological structure of PRRSV GP5 consists of two ectodomains (Ecto-1 and Ecto-2) and a cytoplasmic domain (Cytodomain). (F) HEK293T cells were cotransfected with HA-Nat9 and different Flag-tagged GP5 deletion mutant expression plasmids. The cell lysates were then immunoprecipitated with an anti-Flag MAb and detected by Western blotting at 24 h posttransfection. (G) To determine whether GP5 is a substrate of Nat9, a DTNB-based *in vitro* N-terminal-acetylation assay was performed. GP5-MP peptides were used as negative-control substrates. WCL, whole-cell lysates. Error bars show SD. *, *P* < 0.05.

To prove our hypothesis, we first checked whether GP5 could interact with Nat9 in transient-transfection experiments. We performed coimmunoprecipitation (co-IP) experiments in HEK293T cells transfected with epitope-tagged GP5 and Nat9. As shown by the results in Fig. 4A, epitope-tagged GP5 and Nat9 were coimmunoprecipitated in HEK293T cells. Consistent with this observation, our immunostaining assays showed that GP5 colocalized with Nat9 in transfected HEK293T cells (Fig. 4B).

Nat9 comprises an acetyltransferase domain (amino acids [aa] 33 to 167), a 33-amino-acid N-terminal domain (aa 1 to 33), and a 46-amino-acid C-terminal domain (aa 167 to 212) (Fig. 4C). To determine which domain(s) of Nat9 was required for its interaction with GP5, we created two truncate variants and evaluated them in coimmunoprecipitation experiments (Fig. 4C). We found that both full-length Nat9 and its mutants could interact with GP5 (Fig. 4D). These data suggested that the acetyltransferase domain of Nat9 was required for its interaction with GP5.

GP5 is composed of an N-terminal signal peptide (SP), two ectodomains, two transmembrane domains (TMs), and a C-terminal cytoplasmic domain (Fig. 4E). We next examined which domain(s) of GP5 was required for its interaction with Nat9. Interestingly, the GP5-C truncated mutant (aa 126 to 200), in which the N-terminal ectodomains and the transmembrane domains were deleted, failed to interact with Nat9 (Fig. 4F). In contrast, the GP5-N truncated mutant (aa 1 to 88), which contained the first ectodomain, had little effect on its interaction with Nat9 (Fig. 4F). These results suggested that the first ectodomain of GP5 was required for its interaction with Nat9.

To further test whether Nat9 targeted GP5 for N-terminal acetylation, we used synthesized GP5 N-terminal peptides with M-L, M-R, or M-K as the first two residues and performed an *in vitro* DTNB [5,5′-dithiobis-(2-nitrobenzoic acid)] assay (Fig. S1C) (27). We found that GP5-M-L and GP5-M-R, but not GP5-M-K, were directly acetylated by Nat9 (Fig. 4G). This finding provides direct biochemical evidence suggesting that Nat9 promotes N-terminal acetylation of GP5.

Taken together, these findings suggest that Nat9 interacts with the first ectodomain of GP5 through its main acetyltransferase domain and that the interaction brings Nat9 colocalization with GP5 and promotes N-terminal acetylation of GP5 by Nat9.

### Nat9-mediated N-terminal acetylation promotes GP5 proteolysis but does not affect its ER localization.

**FIG 5:**
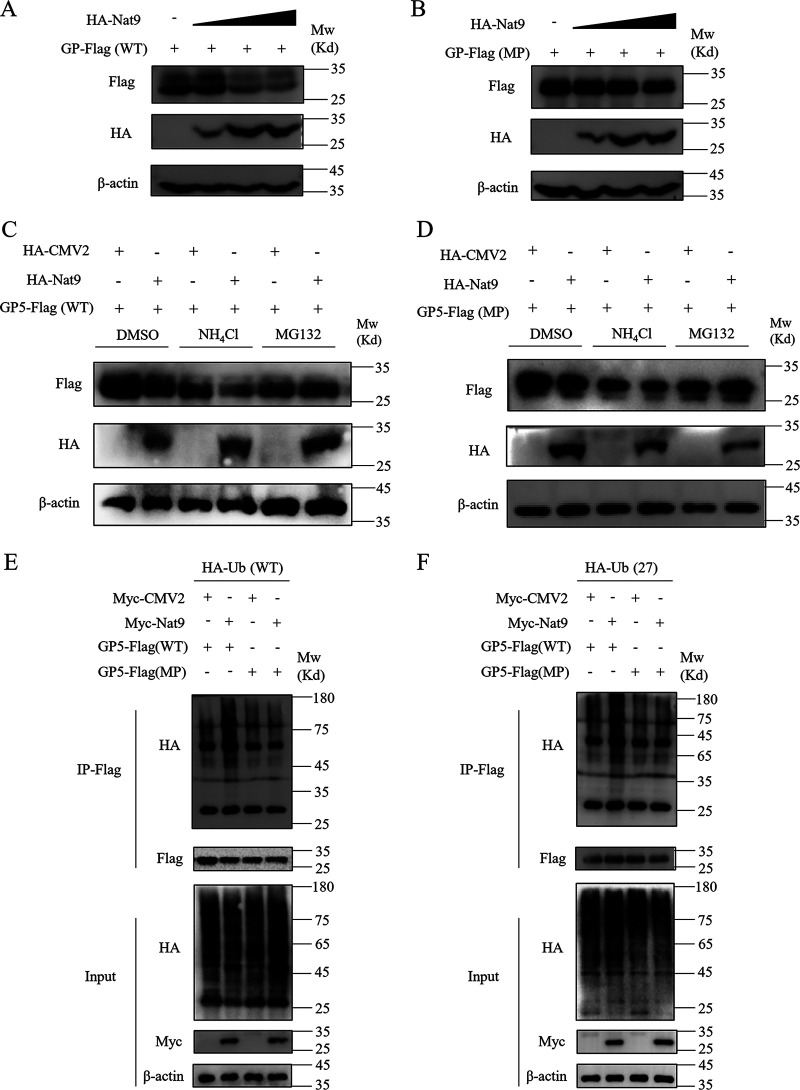
Nat9 mediated the degradation of GP5 through the proteasome pathway. (A, B) HEK293T cells were cotransfected with HA-Nat9 plasmid (HA-Nat9 at 100 ng, 500 ng, and 1,000 ng) and GP5-Flag (WT) plasmid (A) or GP5-Flag (MP) plasmid (B). After 24 h posttransfection, cell lysate supernatants were collected and Western blotting was performed. (C, D) HEK293T cells were cotransfected with HA-Nat9 and GP5-Flag (WT) plasmid (C) or GP5-Flag (MP) plasmid (D) for 12 h and then treated with MG132 (5 μM), NH_4_Cl (5 μM), or dimethyl sulfoxide (DMSO) for 4 h. The protein expression of GP5 was tested by Western blotting. (E, F) HEK293T cells were cotransfected with Myc-Nat9 and GP5-Flag (WT) or GP5-Flag (MP) plasmid, followed by HA-Ub (WT) plasmid (E) or HA-Ub (27) plasmid (F). At 24 h posttransfection, the ubiquitin level of GP5 was detected by immunoprecipitation with the anti-Flag beads and IB with HA antibody.

As GP5 is the major capsid glycoprotein of PRRSV and it is necessary for the infectivity and assembly of PRRSV (28), we wondered how the N-terminal acetylation influences GP5’s function. N-terminal acetylation has been suggested to be an important regulator of protein functions, such as promoting protein proteolysis and inhibiting protein translocation into the endoplasmic reticulum (ER) (24). We first explored whether N-terminal acetylation impacts GP5’s stability in the transient-transfection experiments. As shown by the results in Fig. 5A, the protein expression level of GP5 decreased in a dose-dependent manner after overexpression of Nat9. To confirm whether N-terminal acetylation modification is involved in the GP5 protein stability, we constructed a mutant with the second amino acid, leucine, changed to proline (GP5-MP), which would not be N-terminally acetylated, as previously reported (24). As expected, the protein expression level of GP5-MP was unaffected even with the high dose of Nat9 (Fig. 5B). Consistent with this, the proteasome inhibitor (MG132), rather than the lysosome inhibitor (NH_4_Cl), blocked the degradation of GP5 promoted by Nat9 (Fig. 5C and D), suggesting that Nat9-mediated N-terminal acetylation triggered the degradation of GP5 through the proteasome pathway.

As ubiquitination is a common modification responsible for the proteasome-dependent degradation of proteins, we next tested whether Nat9-mediated N-terminal acetylation regulates GP5 function by affecting GP5 ubiquitination. We transfected ectopically tagged GP5-WT or GP5-MP and Nat9 with wild-type ubiquitin and performed *in vivo* ubiquitination assays in HEK293T cells. As shown by the results in Fig. 5E, GP5-WT and GP5-MP were both polyubiquitinated when cells were cotransfected with a plasmid expressing wild-type ubiquitin, while the ubiquitination of GP5-WT, but not GP5-MP, was enhanced with Nat9 coexpression, suggesting that Nat9 plays a specific role in regulating the ubiquitination of GP5-WT in an N-terminal-acetylation-dependent manner. To distinguish which type of lysine linkage (K27, K48, or K63) is required for N-terminal-acetylation-triggered degradation of GP5, we employed ubiquitin mutants in which only one lysine (at position K27, K48, or K63) was available for ubiquitination. As shown by the results in Fig. 5F and Fig. S2A and B, the K27-linked ubiquitination of GP5-WT was markedly increased by coexpression of Nat9, whereas the K48-linked and K63-linked ubiquitination of GP5-WT and the K27-linked ubiquitination of GP5-MP were not influenced under the same conditions. These findings suggest that Nat9-mediated N-terminal acetylation triggers K27-linked ubiquitination of GP5 and promotes its proteolysis.

Besides acting as a protein degradation signal, N-terminal acetylation has also been indicated to be an inhibitor of endoplasmic reticulum translocation (29). As a capsid glycoprotein, it is important for GP5 to translocate from the ER to the Golgi apparatus, where posttranslational modifications, such as glycosylation, are made during the proliferation of PRRSV. We next asked whether Nat9-mediated N-terminal acetylation of GP5 affects its cellular localization in HeLa cells. As the cellular localization of porcine Nat9 is not fully understood, we first transfected ectopically tagged Nat9 together with empty vector, green fluorescent protein (GFP)-ER, GFP-Golgi, or GFP-lysosome (30) into HeLa cells. We found that porcine Nat9 was mainly localized both in the nucleus and the cytoplasm, and it was interesting that porcine Nat9 was partially colocalized with the GFP-ER marker (Fig. S3A). To our surprise, overexpressed GP5-WT was colocalized with mCherry-ER in HEK293T cells, whereas GP5-Flag (MP) still colocalized with mCherry-ER in HeLa cells (Fig. S3B), implying that N-terminal acetylation by Nat9 did not inhibit the ER localization of GP5. To further sustain our hypothesis, we transfected GP5-WT together with Nat9-WT, Nat9-AAA, or Nat9-ΔAc, and we found that all of the Nat9 variants colocalized with GP5-WT and mCherry-ER in HeLa cells (Fig. S3C). These findings suggest that Nat9 colocalized and interacted with GP5 but the N-terminal acetylation by Nat9 did not affect the ER localization of GP5.

Above all, these findings suggest that Nat9-mediated N-terminal acetylation promotes GP5 proteolysis in a K27-linked-ubiquitination-dependent manner but does not affect its ER localization.

### PRRSV antagonizes Nat9 restriction by inhibiting its transcriptional level through Nsp1β, Nsp4, Nsp9, and N.

**FIG 6:**
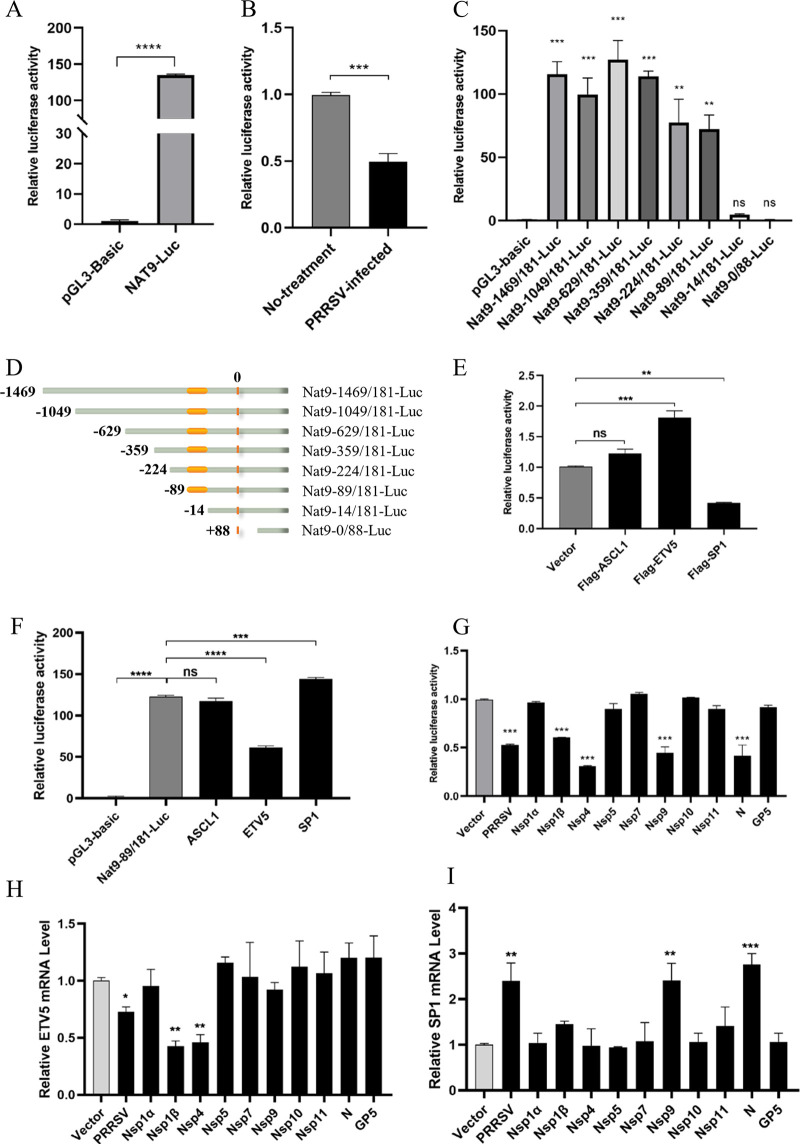
PRRSV inhibits Nat9 expression by regulating the transcription of ETV5 and SP1. (A) A 1,650-bp Nat9 promoter was cloned into the pGL3-basic vector, and the luciferase activity was measured. (B) HEK293T cells were cotransfected with Nat9-Luc and pRL-TK and infected with PRRSV (MOI = 0.5) or not. Luciferase activity was measured using the supernatants of cell lysates. pGL3-Basic was the negative control. (C) HEK293T cells were cotransfected with Nat9 luciferase mutant expression plasmids and pRL-TK, and luciferase activity was measured using the supernatants of cell lysates. (D) Schematic diagram of the Nat9 core promoter region. (E) HEK293T cells were cotransfected with a series of Nat9 promoter binding site mutants and the pRL-TK *Renilla* luciferase reporter plasmid. After 24 h, the luciferase activity was measured. (F) Flag-ASCL1, Flag-ETV5, Flag-SP1, and Flag-CMV2 plasmids were cotransfected with Nat9-89/181-Luc and the pRL-TK *Renilla* luciferase reporter plasmid. After 24 h, the luciferase activity was measured. (G) HEK293T cells were cotransfected with a series of plasmids encoding PRRSV proteins (Nsp1α, Nsp1β, Nsp4, Nsp5, Nsp7, Nsp9, Nsp10, Nsp11, N, and GP5) or empty vector, Nat9-89/181-Luc, and the pRL-TK *Renilla* luciferase reporter plasmid. After 24 h posttransfection, the cells were harvested for luciferase activity analysis. (H, I) HEK293T cells were transfected with plasmid encoding the PRRSV infectious clone or eukaryotic expression plasmids encoding PRRSV genes. At 24 h posttransfection, ETV5 and SP1 expression levels were detected by qRT-PCR. Error bars show SD. *, *P* < 0.05; **, *P* < 0.01 (analysis of one-way ANOVA followed by Bonferroni post-test). Data are representative of three independent experiments.

PRRSV has developed a variety of complex mechanisms to escape the host innate immunity, including interrupting the regulation of host transcription factors (23, 31, 32). As we have proved in our above-described results (Fig. 1), both the mRNA and protein levels of Nat9 were decreased after PRRSV infection. To clarify the molecular mechanism of PRRSV’s transcriptional regulation of Nat9, we first decided to map the core promoter region and identify transcriptional regulators of Nat9. DNA sequences located between −1469 and +181 bp from the Nat9 transcription start site (TSS) were cloned from 3D4/21 cells and used to make constructs in the pGL3-basic plasmid for further promoter activity assays in HEK293T cells. The luciferase activities of the sequences from −1469 to +181 bp from the Nat9 TSS were significantly increased compared to the luciferase activity with an empty pGL3-basic vector (Fig. 6A), and decreased promoter activity after PRRSV infection was further verified (Fig. 6B). To identify the core promoter regions, which are usually composed of 50- to 200-bp DNA fragments that ensure the normal transcription initiation of RNA polymerase II (33), a series of truncated mutants were constructed and the luciferase activities of the truncated mutants were measured. As shown in Fig. 6C and D, the DNA fragment from −89 to −14 bp from the TSS was indicated to be the core promoter region of Nat9 that was essential for the luciferase activity of the truncated mutants.

To identify the transcriptional regulators of Nat9, the core promoter region of Nat9 was analyzed with bioinformatic tools (https://jaspar.genereg.net/), and three transcription factors, ASCL1, ETV5, and SP1, were predicted to bind with this region (Table 1 and Fig. S4A). To further confirm whether these predicted transcription factors were involved in the regulation of Nat9 transcription, we mutated the putative binding sites of ASCL1, ETV5, and SP1 of Nat9-89/181-Luc (Fig. S4B) and checked whether Nat9’s core promoter activity was influenced. As shown by the results in Fig. 6E, mutation of ETV5’s or SP1’s putative binding site dramatically affected Nat9’s core promoter activity, whereas mutation of ASCL1’s putative binding site had no such influence. Consistent with this, the activity of the Nat9 core promoter was significantly increased in ETV5-overexpressed cells and inhibited in SP1-overexpressed cells, while the overexpression of ASCL1 did not affect the activity of the Nat9 core promoter (Fig. 6F). In addition, the activity of the Nat9 core promoter was increased in a dose-dependent manner after overexpression of ETV5 (Fig. S4C), whereas the overexpression of SP1 produced the opposite results (Fig. S4D). These results suggested that ETV5 and SP1 are important transcription factors for the regulation of Nat9’s core promoter activity, and ETV5 is identified as an activator, whereas SP1 is identified as a suppressor.

**TABLE 1:** The predicted transcription factors binding to the core promoter region

Model ID^*a*^	Model name	Score	Relative score	Position		Strand	Predicted site sequence
				Start	End		
MA0079.1	SP1	10.3807	0.948958176078	46	55	+	AGGGCGGGGT
MA0765.1	ETV5	8.28388	0.883691851912	38	47	+	GGCGGAAGAG
MA1100.2	ASCL1	7.97163	0.881878767455	20	29	+	GGCGGCTGCC

a ID, identification number.

To explore whether PRRSV inhibits Nat9 transcription by regulating the transcription factors of the Nat9 core promoter region, we further determined the effects of PRRSV and its proteins on Nat9’s core promoter activity and the mRNA levels of ETV5 and SP1. Nat9’s core promoter activity was inhibited by overexpression of the Nsp1β, Nsp4, Nsp9, and N proteins, as well as by PRRSV infection (Fig. 6G), whereas the mRNA level of the transcriptional activator, ETV5, was inhibited by overexpression of the Nsp1β and Nsp4 proteins (Fig. 6H) and the mRNA level of the transcriptional suppressor, SP1, was enhanced by overexpression of Nsp9 and N proteins (Fig. 6I). In addition, the transcription and protein levels of the N and Nsp2 genes of PRRSV were significantly downregulated under overexpression of ETV5 (Fig. S4E and G), whereas the overexpression of SP1 produced opposite results (Fig. S4F and H). Above all, these results indicate that Nsp1β and Nsp4 of PRRSV downregulate the mRNA level of ETV5 and Nsp9 and N of PRRSV upregulate the mRNA level of SP1, which collaboratively inhibits the transcription of Nat9.

## DISCUSSION

PRRSV infection has caused significant economic losses to the pig industry worldwide since it first emerged in the late 1980s from Europe and North America (34). It is important to explore the molecular mechanisms of PRRSV’s pathogenesis and the host defense response to help prevent disease and reduce losses. Dynamic transcriptional regulation and posttranslational modifications play important roles in the process of PRRSV infection and in host antiviral immunity. For example, PRRSV upregulates the transcriptional level of TFDP2 to positively regulate cyclin A expression and trigger a smaller proportion of cells in the S phase, which contributes to PRRSV proliferation (23). Posttranslational modifications like ubiquitination and deubiquitination modification have been proven to affect the stability and functions of host proteins and impair the proliferation of PRRSV (35, 36). Other posttranslational modifications, such as glycosylation, may also play important roles in PRRSV proliferation, but further investigations on the molecular mechanisms are required (37). In this study, we focused on the N-acetyltransferase Nat9, whose transcriptional level was dramatically decreased after PRRSV infection in our transcriptome analysis of PRRSV-infected PAM cells (38, 39). The N-terminal acetylation modification of PRRSV, to our knowledge, has not been reported so far, despite the fact that N-terminal acetylation has been suggested to play important roles in the process of some viruses’ infection and replication, such as influenza virus, enterovirus 71 and L-A double-stranded RNA virus (19, 21, 40, 41).

We initially demonstrated that the mRNA and protein levels of Nat9 were dramatically decreased after PRRSV infection, indicating that PRRSV may influence Nat9-mediated posttranslational modification during its proliferation. Next, we showed that overexpression of Nat9 inhibited PRRSV replication, while knockdown and knockout of Nat9 enhanced PRRSV replication. Meanwhile, the overexpression of Nat9 wild type, but not Nat9-AAA or Nat9-ΔAc, could rescue the phenomena in Nat9-KO cells compared with Nat9-WT. These findings suggest that Nat9 is a host restriction factor for PRRSV proliferation and that the N-acetyltransferase activity is required for its inhibitory function.

A previous study reports that histone deacetylase 6 (HDAC6) overexpression suppresses viral gene expression and PRRSV production in virus-challenged PAM cells and that HDAC6 might exert its antiviral activity by suppressing the level of acetylated α-tubulin in PRRSV-infected cells (42); however, whether viral proteins could be acetylation modified has not been reported. N-terminal acetylation is processed by NAT family members with a dependence on the first two amino acids, and M-L or M-R is recognized by Nat9 as the substrate feature (26). To test whether viral proteins could be modified by N-terminal acetylation, we analyzed the amino acid sequences of the PRRSV genes, and only the GP5 protein contained M-L as the featured sequence of Nat9. More importantly, although GP5 is the most variable structural protein of PRRSV and the N-terminal amino acid sequence is not conserved (43), M-L or M-R is the major amino acid feature of the GP5 protein in almost 90% of the PRRSV genomes we have analyzed. Consistent with this, we found that Nat9 could interact with GP5 and that synthesized GP5 N-terminal peptides with M-L or M-R were directly acetylated by Nat9 *in vitro* in a DTNB [5,5′-dithiobis-(2-nitrobenzoic acid)] assay. For those PRRSV strains without the M-L or M-R featured sequence, classical NAT family members, such as NatA, NatB, NatC, and NatE, were predicated to carry out the N-terminal-acetylation modification according to the relevant featured sequence recognized by the NATs (25). These findings suggest that Nat9 is responsible for the N-terminal-acetylation modification of the GP5 protein of almost 90% of PRRSV strains with the M-L or M-R featured sequence and that this may be an important universal host modification for combating PRRSV infection.

To further clarify the molecular regulation mechanism of Nat9 on PRRSV proliferation, we next determined the effect of the N-terminal-acetylation modification on the protein stability and subcellular localization of GP5. We confirmed that Nat9-mediated N-terminal acetylation of GP5 promoted its protein degradation in a proteasome-dependent manner and that K27-linked ubiquitin modification was enhanced and responsible for the proteasome degradation, whereas Nat9-mediated N-terminal acetylation did not affect the ER residency of GP5, as GP5 colocalized with either Nat9 wild type (Nat9-WT) or N-acetyltransferase-inactive mutants (Nat9-AAA or Nat9-ΔAc) in the ER. However, the E3 ubiquitin ligase responsible for K27-linked ubiquitination of GP5 needs to be further identified, and whether the universal host-mediated N-terminal acetylation of GP5 affects the assembly of virion particles is worth further exploration.

PRRSV has evolved and employed various kinds of weapons to antagonize the host defense systems, and disruption of host transcription factors to evade innate immune responses is a common mechanism. Both the nonstructural proteins and structural proteins have been found to regulate different host transcription factors to favor PRRSV proliferation (31, 32, 44–46). In this study, we characterized the sequence from −89 to −14 bp upstream from the transcription start site (TSS) as the core promoter of Nat9 by using luciferase assay experiments and found that ETV5 acts as an activator and SP1 acts as a suppressor for the transcription of the Nat9 gene through binding to this region. To better understand how PRRSV regulates the transcription of Nat9, we screened the influence of overexpressed nonstructural and structural proteins of PRRSV on the mRNA levels of ETV5 and SP1 in our library. We found that Nsp1β and Nsp4 downregulate the mRNA levels of ETV5 and Nsp9 and N upregulates the mRNA level of SP1, which collaboratively inhibits the transcription of Nat9. Considering that N-terminal acetylation is a universal modification of PRRSV and the mRNA levels of most family members of NATs were changed in our transcriptome analysis, we believe that disrupting the expression of NAT family members via PPRSV proteins must be an important manner of evading the host defense system.

**FIG 7:**
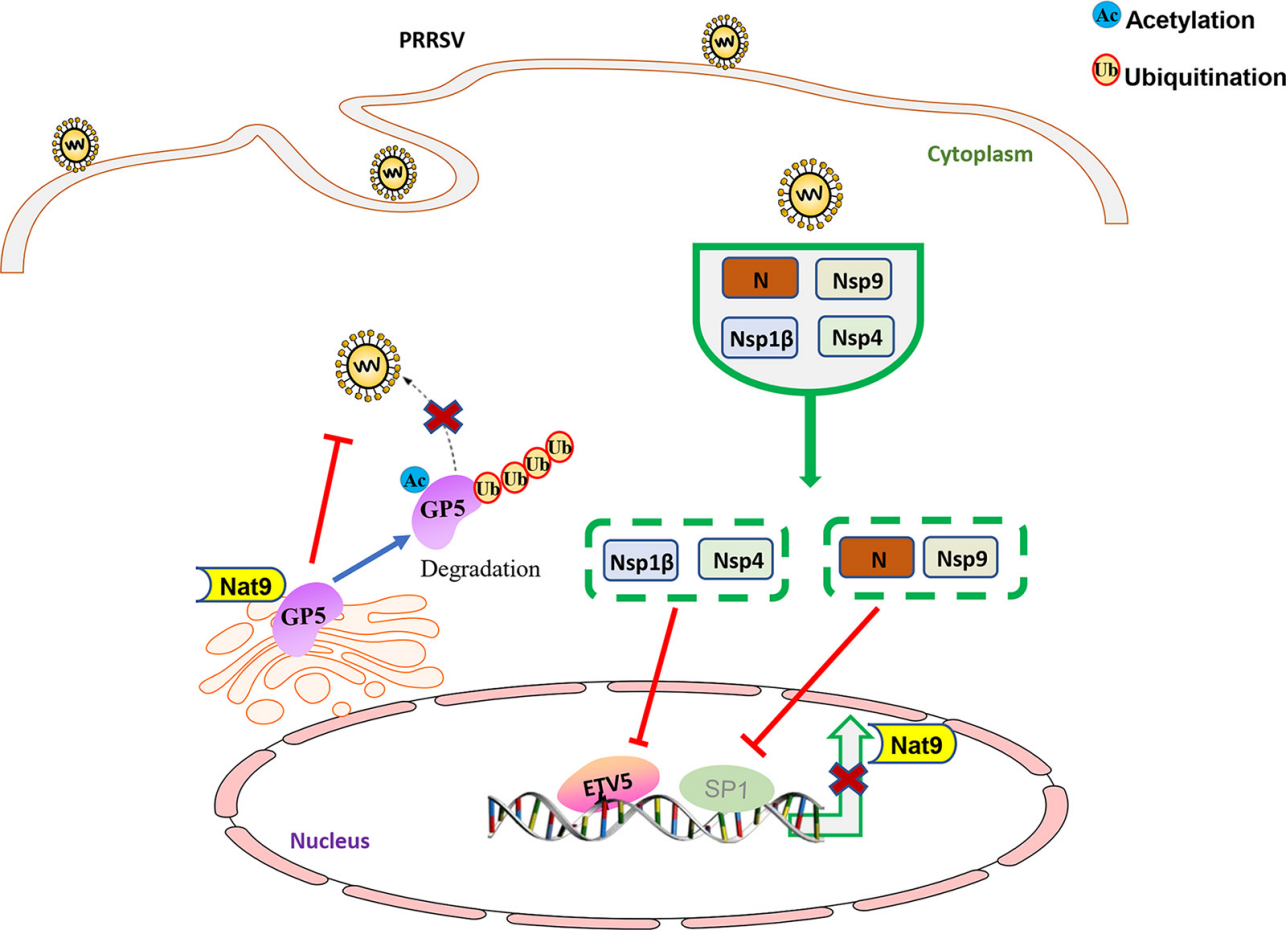
Diagram of the mechanism of Nat9’s inhibition of PRRSV proliferation. After PRRSV infection, Nat9 acetylates the N terminus of GP5 and generates a degradation signal, which degrades GP5 though the proteasome pathway and affects virion assembly. PRRSV regulates transcription factors ETV5 and SP1 through its Nsp1β, Nsp4, Nsp9, and N proteins, thereby regulating the transcription activity of Nat9 and inhibiting the expression of Nat9.

In summary, we demonstrated N-terminal acetylation by Nat9 as a novel host defense mechanism that leads to K27-linked-ubiquitination-dependent proteolysis of GP5, which contributes to inhibiting PRRSV infection and proliferation in 3D4/21 cells. Meanwhile, PRRSV antagonizes Nat9’s restriction by inhibiting its transcriptional level through Nsp1β, Nsp4, Nsp9, and N, which is important for understanding the molecular and cellular mechanisms of PRRSV pathogenesis (Fig. 7).

## MATERIALS AND METHODS

### Cells and virus.

Porcine alveolar macrophage (PAM) cell line CRL2843-CD163 (3D4/21 cells) was maintained in RPMI 1640 medium (Gibco, Carlsbad, CA, USA) supplemented with 10% (vol/vol) fetal bovine serum (FBS; Biological Industries, Kibbutz Beit-Haemek, Israel), penicillin (100 U/mL), and streptomycin (100 mg/mL). Human embryonic kidney HEK293T cells and HeLa cells were cultured in Dulbecco’s modified Eagle’s medium (DMEM; Gibco) supplemented with 10% FBS. All cells were maintained at 37°C with 5% CO_2_. The Chinese highly pathogenic PRRSV strain JXwn06 was used in this study (22, 23).

### Antibodies and reagents.

Anti-PRRSV Nsp2 polyclonal antibody (PAb) and anti-N monoclonal antibody (MAb) were kindly contributed by Jun Han from China Agricultural University. Anti-Nat9 PAb was prepared by immunizing mice with the purified protein. Mouse anti-Flag MAb (30503ES60; Yeasen), anti-Flag-horseradish peroxidase (HRP) Ab (A8592; Sigma-Aldrich), rabbit antihemagglutinin (HA) MAb (3724S; CST), mouse anti-Myc MAb (HT101; TransGen), rabbit anti-β-actin PAb (30102ES40; Yeasen), anti-mouse IgG-HRP-linked antibody (7076S; CST), anti-rabbit IgG-HRP-linked antibody (7074; CST), goat anti-mouse IgG Alexa Fluor 647 (33213ES60; Yeasen), goat anti-mouse IgG Alexa Fluor 555 (A21422; Invitrogen), goat anti-rabbit IgG Alexa Fluor 555 (A21428; Invitrogen), fluorescein isothiocyanate (FITC)-conjugated anti-mouse IgG (86603S; CST), and mouse anti-Flag beads (Abmart) were used.

### Plasmid constructs.

The cDNA encoding Nat9 was amplified from porcine peripheral blood mononuclear cells (PBMCs) and cloned into vectors pFlag-CMV2/pHA-CMV2/pMyc-CMV2/pEGFP-c1 to construct a recombinant plasmid by seamless cloning technology. cDNA encoding full-length nonstructural protein (Nsp) or structural protein from PRRSV strain JXwn06 was subcloned into expression vector pFlag-CMV2 or pcDNA3.1-A(-)-Flag. Nat9 luciferase reporter plasmids were constructed. The primers used are shown in Table 2.

**TABLE 2:** Primers used in PCR amplification

Primer	Sequence (5′–3′)
pCMV-Nat9	F: CAAGCTTGCGGCCGCGAATTCATGCTGCAGGCTGCTACCAT
	R: TGCCACCCGGGATCCTCTAGATCAGTGGGGCTCTGAGGACC
pEGFP-Nat9	F: TCGAGCTCAAGCTTCGAATTCTATGCTGCAGGCTGCTACCAT
	R: GGATCCCGGGCCCGCGGTACCTCAGTGGGGCTCTGAGGACC
pMal-c5x-Nat9	F: CGCGATATCGTCGACGGATCCATGCTGCAGGCTGCTACCAT
	R: ACCTGCAGGGAATTCGGATCCTCAGTGGGGCTCTGAGGACC
pCMV-Nat9 (1-167)	F: CTGCACTTTGAGTGATCTAGAGGATCCC
	R: CTCAAAGTGCAGCCTGCGGA
pCMV-Nat9 (33-212)	F: GCCGCGAATTCACATGAGTGGATGAAA
	R: TGAATTCGCGGCCGCAAGA
GP5-Flag	F: CCACACTGGACTAGTGGATCCGCCACCATGTTGGGGAAGTGCTTGAC
	R: CTTGGTACCGAGCTCGGATCCGAGACGACCCCATTGTTCCG
GP5-Flag (1-88)	F: CAGCCATTTCCTTAAGCTTGGGCCCGACTAC
	R: AAGGAAATGGCTGGTGGTGA
GP5-Flag (126-200)	F: ATCCGCCACCATGAGGCTTGCGAAGAACTGC
	R: CATGGTGGCGGATCCACTAG
Nat9-1469/181-Luc	F: ATCTGCGATCTAAGTAAGCTTTCTTACGCCATCCAAGACTAT
	R: CAGTACCGGAATGCCAAGCTTGAGACAGGCCCAAGGCCTC
Nat9-1049/181-Luc	F: CTAAGTAAGCTTCTCCGGTCTCGGTGA
	R: AAGCTTACTTAGATCGCAGATCTCGA
Nat9-629/181-Luc	F: CTAAGTAAGCTTCACTACAAGGGTCCCCT
	R: AAGCTTACTTAGATCGCAGATCTCGA
Nat9-359/181-Luc	F: CTAAGTAAGCTTTCCCCCCTCCTAGAGAA
	R: AAGCTTACTTAGATCGCAGATCTCGA
Nat9-224/181-Luc	F: CTAAGTAAGCTTCAGCTGTCCCGCAC
	R: AAGCTTACTTAGATCGCAGATCTCGA
Nat9-89/181-Luc	F: CTAAGTAAGCTTGGGACCGGGATTGG
	R: AAGCTTACTTAGATCGCAGATCTCGA
Nat9-14/181-Luc	F: CTAAGTAAGCTTGAGGAAAGGCCGTC
	R: AAGCTTACTTAGATCGCAGATCTCGA
Nat9-0/88-Luc	F: CTAAGTAAGCTTCCCGGGGCTTCAGT
	R: AAGCTTACTTAGATCGCAGATCTCGA
Nat9-ETV5-Mutant -Luc	F: GTGGCAATAGAGGGCGGGGTGCG
	R: CCCTCTATTGCCACGCAGACGGCA
Nat9-SP1-Mutant -Luc	F: GAAGAGATCGGGGTGCGGTGCTACG
	R: CCCCGTACTCTTCCGCCACGCAGAC
Nat9-ASCL1-Mutant-Luc	F: CCGGTGGCAACCGTCTGCGTGGCGG
	R: CGGTTGCCACCGGGGCCCAATCCCG

### Lentivirus production and generation of stable cell lines.

Nat9-deficient HEK293T cells were generated by using the clustered regularly interspaced short palindromic repeat (CRISPR)/Cas9 technology. A single guide RNA (sgRNA) targeting Nat9 was cloned into a Cas9-expressing lentiviral transfer vector (lentiCRISPRv2). Briefly, custom-designed mutant primers (Nat9-sgRNA) oriented in the inverse direction were used to amplify the entire circular template of lentiCRISPRv2. The lentiCRISPRv2 plasmid was cotransfected into HEK293T cells with packaging vectors pMD2G and psPAX2 by using liposomal transfection reagent (Yeasen, Shanghai, China). Two days after transfection, the viral supernatant was harvested and stored at −80°C. 3D4/21 cells were incubated with virus supernatant containing 8 mg/mL Polybrene for 48 h and selected with 4 μg/mL puromycin to generate a stable cell line. The oligonucleotides for sgRNAs were as follows: forward, 5′-*GGCACATGCTCCGGGGTGTA*GTTTTAGAGCTAGAAATAGCAAGT-3′, and reverse, 5′-*TACACCCCGGAGCATGTGCC*GGTGTTTCGTCCTTTCCACAAGATAT-3′.

### Transcriptome sequencing and analysis.

3D4/21 cells were grown in 6-well plates at an initial concentration of 10^6^ cells/mL and were then inoculated with PRRSV at a multiplicity of infection (MOI) of 0.5 for 24 h. Virus-infected cells were washed twice with cold phosphate-buffered saline (PBS), and 1 mL of TRIzol reagent was added. The treated cells were used for transcriptional sequencing by the Guangzhou Gene Denovo Biotechnology Company. The transcriptome data obtained and a heat map of differentially expressed genes were analyzed using GraphPad Prism 8.

### qRT-PCR.

Quantification of the relative levels of gene expression was performed using quantitative reverse transcription-PCR (qRT-PCR). Total RNA from 3D4/21 cells was extracted using TRIzol reagent (TaKaRa), and the first-strand cDNA was synthesized using a first-strand synthesis system (TransGen, China) according to the manufacturer’s instructions. The relative levels of gene expression were analyzed by using the qTower3 G *in vitro* diagnostic (IVD) fluorescence quantitative PCR system (Jena, Germany) using TransStart top green qPCR super mix (TransGene) with three-step amplification. The data were calculated based on the cycle threshold (2^−ΔΔ^*^CT^*) method, and the data were normalized to the expression level of the β-actin gene. All of the primer pairs used for quantitative real-time PCR are listed in Table 3.

**TABLE 3:** Primers used in quantitative real-time PCR

Gene	Accession no.	Primer sequence (5′–3′)
β-Actin	DQ452569.1	F: GAATCCTGCGGCATCCACGA
		R: CTCGTCGTACTCCTGCTTGCT
qNat9	XM_005668615.3	F: AGGCTGCTACCATGAGGTTA
		R: CTCTGCTGCATCGCGTACT
PRRSV-N	ABR37297.1	F: CAGTCAATCAGCTGTGCCAAA
		R: ATCTGACAGGGCACAAGTTCCA
PRRSV-Nsp2	ABR37297.1	F: CAGCCTTATGACCCCAACCAG
		R: TGGGCAAAGTCCCCTGTACCAA
qETV5	NM_001243025.1	F: GTGTCGTTCCTGAAAGACTGGA
		R: CGGCCTGTCCAGGCAATGAAGT
qSP1	XM_005652567.3	F: ACGCTTCACACGTTCGGATGAG
		R: TGACAGGTGGTCACTTCTCATG

### Western blot analyses.

The transfected or virus-infected cells were lysed in lysis buffer (50 mM Tris-HCl [pH 7.5], 100 mM NaCl, 0.5% Triton X-100, 10% glycerol, 1 mM EDTA) supplemented with a proteinase inhibitor (20 nM phenylmethylsulfonyl fluoride [PMSF]; Sigma-Aldrich). Cells lysis supernatants were measured and boiled in the buffer for 10 min. Equal amounts of extracts were separated with SDS-PAGE and transferred to methanol-activated polyvinylidene difluoride (PVDF) membranes (Millipore). Membranes were blocked with 5% skimmed milk in 1× TBST (Tris-buffered saline with 0.05% Tween 20) for 1 h and incubated overnight at 4°C with an antibody against Nat9 (1:12,000), PRRSV Nsp2 (1:2,000), or PRRSV N (1:1,000) or with labeled Abs (1:2,000). This was followed by washing and incubation with HRP-conjugated antibody for 1 h at room temperature. Immunodetection was completed using Pierce enhanced chemiluminescence (ECL) Western blotting substrate (Thermo Fisher Scientific).

### RNA interference.

3D4/21 cells were transfected with small interfering RNAs (siNat9-1 and siNat9-2) at a final concentration of 50 nM using liposomal transfection reagent (Yeasen). All siRNAs were synthesized by GenePharma (Shanghai, China) (Table 4). The knockdown efficiency was confirmed by qRT-PCR and Western blotting.

**TABLE 4:** Primers used in RNA interference assay

Primer	Sequence (5′–3′)
Negative control	F: UUCUCCGAACGUGUCACGUTT
	R: ACGUGACACGUUCGGAGAATT
siNat9-1	F: GGCUGCUACCAUGAGGUUATT
	R: UAACCUCAUGGUAGCAGCCTT
siNat9-2	F: GCAUGUGCCUAGGUACCAUTT
	R: AUGGUACCUAGGCACAUGCTT

### Immunoprecipitation (IP) and coimmunoprecipitation (co-IP) assays.

HEK293T cells cultivated in six-well plates were transfected or cotransfected with appropriate eukaryotic expression plasmids. The cells were harvested and resuspended in PBS at 24 h posttransfection and lysed on ice for 15 min in 400 μL radioimmunoprecipitation assay (RIPA) lysis buffer. Then, the samples were centrifuged at 12,000 rpm for 5 min. We took out 50-μL amounts of supernatant in new tubes, added 10 μL loading buffer, and boiled the tubes to make input samples. Subsequently, anti-Flag-labeled beads were activated by RIPA and added to the rest of the cell lysis supernatant. The reaction mixture was incubated at 4°C overnight. The beads were washed three times with lysis buffer containing PMSF for 5 min each time, and then 50 μL lysates and 10 μL loading buffer were added and the mixtures boiled for 10 min to make IP samples. Finally, the proteins bound to the beads were separated via SDS-PAGE, transferred to PVDF membranes, and detected with the proper antibodies.

### Confocal immunofluorescence.

HEK293T cells were seeded in glass coverslips in 12-well plates until the cell density was 30% to 40%. Depending on the specific experiment, the plasmids expressing Nat9 (HA-Nat9, HA-Nat9-AAA, HA-Nat9-ΔAc, or enhanced green fluorescent protein [EGFP]-Nat9), GP5 (GP5-Flag-WT or GP5-Flag-MP), Golgi marker (GFP-Golgi), ER marker (GFP-ER or mCherry-ER), LAMP marker (GFP-LAMP1), or empty vector were transfected into HEK293T cells as indicated in the figure legends. In order to detect endogenous immunofluorescence, 3D4/21 cells were infected with 0.5 MOI of PRRSV and incubated at 37°C. At 12 h or 24 h posttransfection or infection, the cells were washed with cold PBS and fixed with 4% paraformaldehyde for 30 min and then permeabilized using 0.3% Triton X-100 in PBS for 15 min at room temperature. The cells were then blocked in 5% bovine serum albumin (BSA) in PBS for 30 min. Next, the cells were incubated with primary Abs (anti-Flag, anti-HA, or anti-Nat9 Ab, diluted at 1:500) in 2% BSA in PBS at 4°C overnight. Next, the cells were washed with PBST buffer (1× PBS and 0.1% Tween 20) and incubated with secondary Ab (anti-mouse IgG Alexa Fluor 647, anti-mouse IgG Alexa Fluor 555, anti-rabbit IgG Alexa Fluor 555, anti-mouse IgG Alexa Fluor 488, or FITC-conjugated anti-mouse IgG, diluted at 1:200) for 1 h. Finally, the cells were washed and stained with DAPI (4′,6-diamidino-2-phenylindole; Solarbio) for 5 min. Imaging of the cells was carried out using a Leica confocal microscope. Images were taken at ×10 or ×100 magnification.

### DTNB *in vitro* N-terminal acetylation assay.

An *in vitro* NAT activity assay was performed under the DTNB protocol (27). DTNB (Ellman’s Reagent, 22582) was purchased from Sigma-Aldrich. The oligopeptides used in this study were custom-made (Synbio Technologies, Suzhou, China): the peptides contained seven specific amino acids at their N termini (GP5-ML, MLGKCLT; GP5-MR, MRCSHKL; GP5-MK, MKCSHRL; and GP5-MP, MPCSHKL), followed by 17 amino acids identical to the adrenocorticotropic hormone peptide sequence (RWGRPVGRRRRPVRVYP).

### Dual-luciferase reporter assay.

HEK293T cells were seeded in 24-well plates and transfected with the constructed plasmids (pRL-TK [20 ng], pGL3-Basic, or Nat9 promoter mutant expression plasmids [200 ng]) using liposomal transfection reagent (Yeasen). At 24 h posttransfection, the cells were infected with or without PRRSV at an MOI of 0.5 for 24 h. The lysed samples were prepared and analyzed for firefly and *Renilla* luciferase activities using a dual-luciferase reporter assay system (Yeasen, Shanghai, China), following the manufacturer’s instructions.

### Determination of virus titer.

The virus titer was determined using the 50% tissue culture infective dose (TCID_50_) method. 3D4/21 cells were seeded in 96-well plates (1 × 10^4^ cells/well), and after the cells adhered to the plate and had grown to about 50% confluence, they were incubated with serial 10-fold dilutions (100 μL) of the primary PRRSV stock, with two replicates of each dilution (1 to 10^−6^) inoculated into the cells. Mock-infected cells were used as controls. The cells were incubated at 37°C for 7 days, and TCID_50_ values were calculated by the Reed-Muench method.

### Statistical analysis.

Statistical analysis was performed using GraphPad Prism 8 software, and statistical significance was calculated by the one-way analysis of variance (ANOVA) method. Results are shown as the mean values ± standard deviations (SD). A *P* value of <0.05 indicates a statistically significant difference.

### Data availability.

The raw data supporting the conclusions of this article will be made available by the authors, without undue reservation.
