# Supplementary material for: N-Acetyltransferase 9 Inhibits Porcine Reproductive and Respiratory Syndrome Virus Proliferation by N-Terminal Acetylation of the Structural Protein GP5
Source: Microbiol Spectr. 2023 Jan 25;11(1):e02442-22. doi: 10.1128/spectrum.02442-22 (PMC9927549; doi:10.1128/spectrum.02442-22)
Supplement: Supplemental file 1 — Supplemental material. Download spectrum.02442-22-s0001.pdf, PDF file, 1.0 MB [file spectrum.02442-22-s0001.pdf]

Supplemental figure legends

Figure S1. The profile of GP5 N-terminal sequence and scheme of the DTNB-based in vitro Nt-acetylation assay.

(A) The N-terminal sequence of PRRSV strains JXwn06 encoded protein. (B) Sequence statistics of GP5 N-terminal from 4081 PRRSV strains. (C) Scheme of the DTNB-based in vitro Nt-acetylation assay.

Figure S2. Detection of K48-, K63-linked polyubiquitination of GP5.

HEK293T cells were co-transfected with Myc-Nat9 and GP5-Flag (WT) or GP5-Flag (MP) plasmid, and following with HA-Ub (63) plasmid (A) or HA-Ub (48) plasmid (B). At 24 h post-transfection, the ubiquitin level of GP5 was detected by immunoprecipitation with the anti-Flag beads and IB with HA antibody.

Figure S3. N-terminal acetylation of GP5 does not affect its endoplasmic reticulum localization

(A) Subcellular localization analysis of porcine Nat9. Hela cells were transfected with expression vectors (GFP-Golgi, GFP-ER or GFP-LAMP1) and HA-Nat9 plasmid. Cells were fixed and permeabilized at 24 h post-transfection. Then cells incubated with a rabbit anti-HA antibody, and attended by Alexa Fluor 555 anti-rabbit IgG (red). Cellular nuclei were stained with DAPI. The porcine Nat9 localization was observed under a laser confocal imaging analysis system, scale bar: 14 μm. (B) GP5-Flag (WT) or GP5-Flag (MP) plasmid and mCherry-ER plasmid were transfected into Hela cells. Cells were fixed and permeabilized at 24 h post-transfection. Then cells incubated with anti-Flag antibody, and followed by FITC-conjugated anti-mouse IgG (green). Cellular nuclei were stained with DAPI. (C) Co-transfection of mCherry-ER with HA-Nat9 (WT), HA-Nat9-AAA or HA-Nat9-△Ac, GP5-Flag (WT) or GP5-Flag (MP) into Hela cells. The cells were fixed and double-stained with a mouse anti-Flag antibody and a rabbit anti-HA antibody and followed by FITC-conjugated anti-rabbit IgG (green) and Alexa Fluor 647 goat anti-mouse IgG (pink). Nuclei were stained with DAPI (blue). Fluorescence confocal microscopy (Leica SP 8, Germany) was used to detect the co-location. scale bar: 14 μm.

Figure S4. ETV5 and SP1 were identified as key transcription factors in porcine Nat9

(A) The positions of the putative transcriptional regulatory elements in the region of Nat9 core promoter were identified. (B) Schematic representation of putative Nat9 promoter binding sites and

the promoter truncated mutants of Nat9 were generated. (C) HEK293T cells were co-transfected with Flag-SP1 (0.1 μg, 0.5 μg, 1 μg), Nat9-89/181-Luc and the pRL-TK Renilla luciferase reporter plasmid, 24 h later, the luciferase activity was measured. (D) HEK293T cells were co-transfected with Flag-ETV5 (0.1 μg, 0.5 μg, 1 μg), Nat9-89/181-Luc and the pRL-TK Renilla luciferase reporter plasmid, 24 h later, the luciferase activity was measured. (E, F) qRT-PCR analysis of N and Nsp2 mRNA in 3D4/21 cells transfected with Flag-ETV5, Flag-SP1 or vector upon PRRSV (MOI=0.5) infection at indicated time. (G, H) Western blot analysis of N and Nsp2 protein leven in 3D4/21 cells transfected with Flag-ETV5, Flag-SP1 or vector upon PRRSV (MOI=0.5) infection at indicated time. *$P < 0.05$, **$P < 0.01$ (analysis of one-way ANOVA followed by Bonferroni post-test). Data are representative of three independent experiments.

Supplementary Figure 1

A

Protein	N-terminus
Nsp1α	MSGILD
Nsp1β	MADVYDI
Nsp2	MAGKRAR
Nsp3	MGPHLIA
Nsp4	MGAFRTQ
Nsp5	MGGLSTV
Nsp6	MGKLREG
Nsp7	MSLTGAL
Nsp8	MAAKLSV
Nsp9	MAASGLT
Nsp10	MGKKSRM
Nsp11	MGSSSPL
Nsp12	MGRHFTW
GP2a	MKEGLCK
GP3	MARQCAR
GP4	MAAPFLF
GP5	**MLGKCLT**
M	MGSLDDF
E	MGSLWSQ
N	MPNNNGK

B

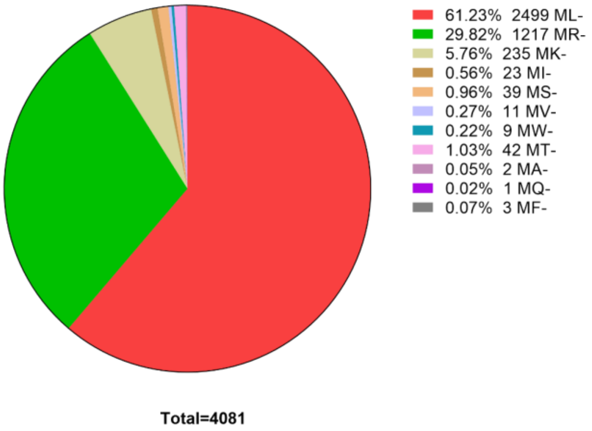

C

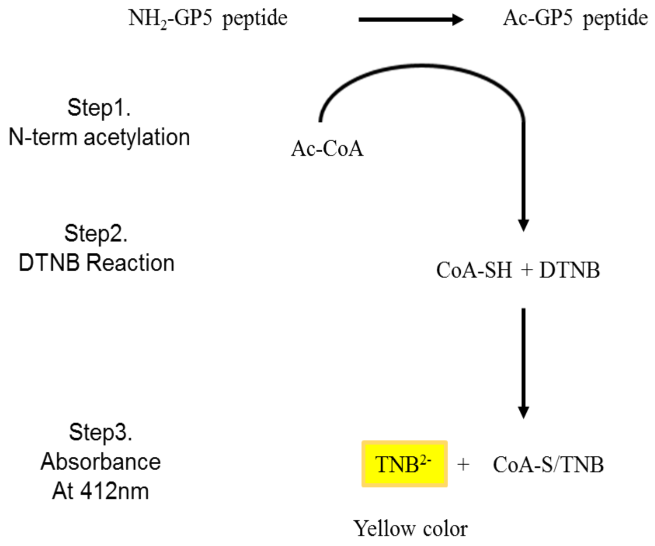

Supplementary Figure 2

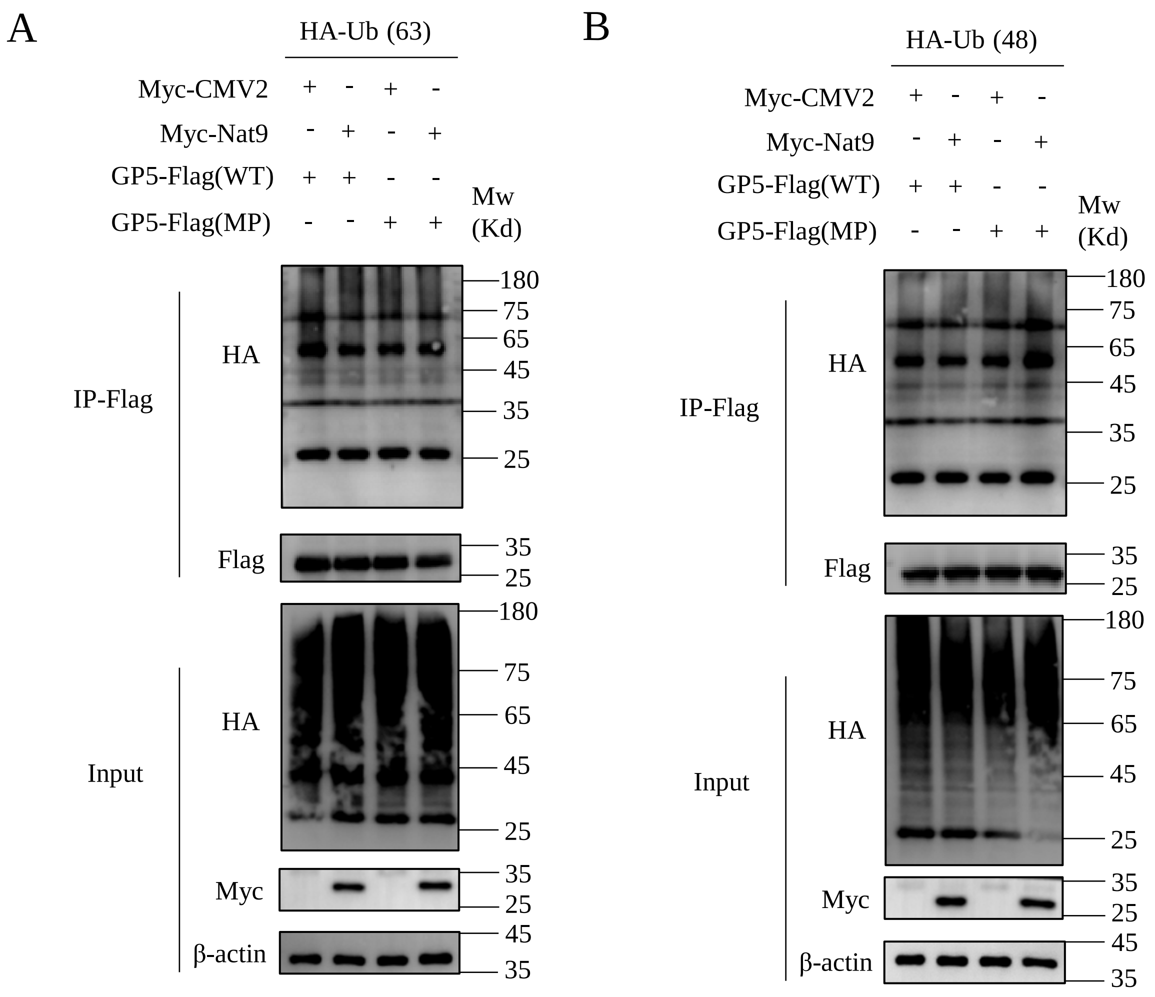

Supplementary Figure 3

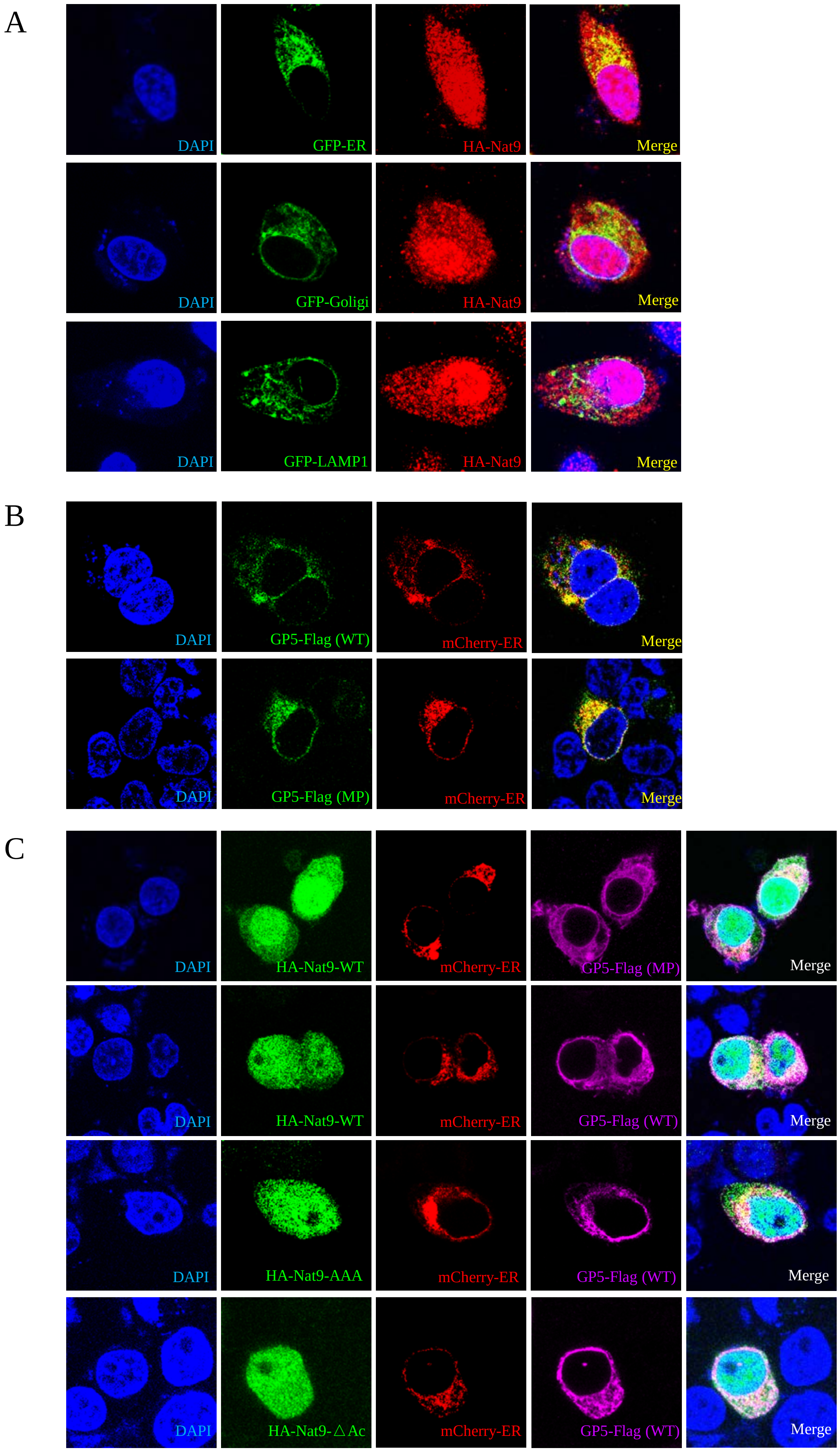

Supplementary Figure 4

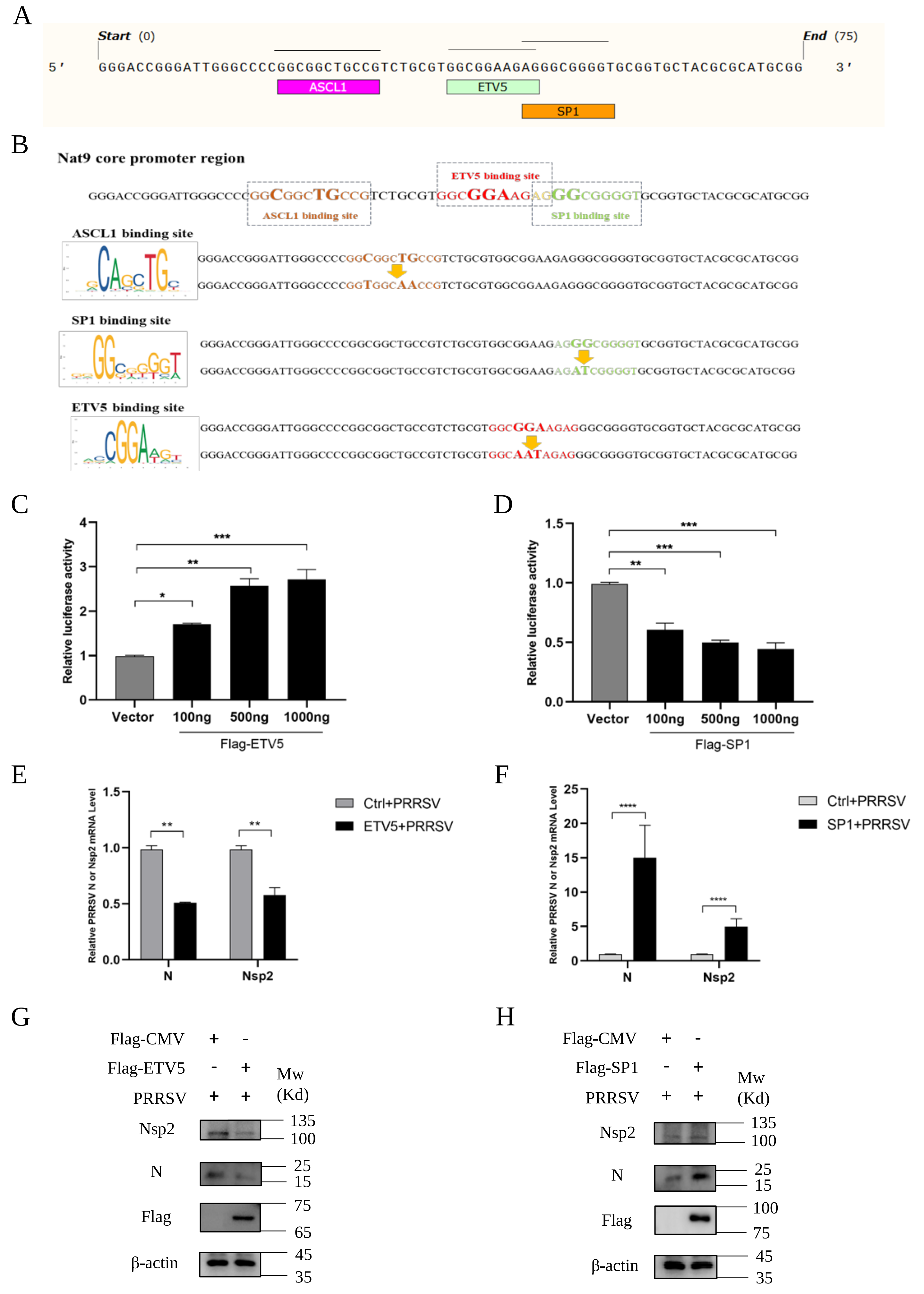

Supplementary Table1. Summary table of the acetylation-related RNA transcripts expression profiles

id	Mock_fpkm	PRRSV_fpkm	log2(FC)	Pvalue	significant	Symbol	Description
rna35643	3.91	1.87	-1.06413	0.00413	yes	ACAT1	acetyl-CoA acetyltransferase, mitochondrial isoform X2
rna30252	0.68	0.001	-9.40939	9.53E-10	yes	BAZ1A	bromodomain adjacent to zinc finger domain protein 1A isoform X1
rna53398	0.001	0.35	8.451211	1.54E-05	yes	BAZ2B	bromodomain adjacent to zinc finger domain protein 2B isoform X11
rna22287	2.79	6.24	1.161281	2.88E-05	yes	BRD1	bromodomain-containing protein 1
rna28889	0.001	2.41	11.23482	3.93E-27	yes	BRD2	bromodomain-containing protein 2 isoform X1
rna10673	0.58	0.001	-9.17991	6.14E-05	yes	BRD8	bromodomain-containing protein 8 isoform X7
rna10916	1.6	3.82	1.255501	0.000115	yes	HDAC3	histone deacetylase 3 isoform X1
rna10916	1.6	3.82	1.255501	0.000115	yes	HDAC3	histone deacetylase 3 isoform X1
rna60449	0.001	1.37	10.41996	1.19E-13	yes	HDAC6	histone deacetylase 6 isoform X2
rna21660	2.78	6.05	1.12185	0.000627	yes	HDAC7	histone deacetylase 7
rna37042	4.49	0.001	-12.1325	1.51E-20	yes	HDAC9	histone deacetylase 9 isoform X4
rna22259	0.001	1	9.965784	4.82E-07	yes	HDAC10	histone deacetylase 10 isoform X1
rna42118	0.43	1.21	1.492598	0.001935	yes	KAT2A	histone acetyltransferase KAT2A isoform X2
rna6444	2.82	6.27	1.15277	3.74E-06	yes	KAT5	K(lysine) acetyltransferase 5
rna50727	0.09	0.85	3.239466	7.41E-10	yes	KAT6B	histone acetyltransferase KAT6B isoform X3
rna42483	0.001	0.55	9.103288	3.07E-05	yes	KAT7	histone acetyltransferase KAT7 isoform X1
rna33863	0.001	3.38	11.72281	1.91E-21	yes	LEF1	lymphoid enhancer-binding factor 1 isoform X1
rna44964	1.44	3.43	1.25214	0.016767	yes	NAT6	N-acetyltransferase 6
rna41461	1.89	0.32	-2.56224	0.004443	yes	NAT9	N-acetyltransferase 9 isoform X2
rna7558	0.77	0.001	-9.58871	3.03E-08	yes	NAT10	N-acetyltransferase 10
rna33495	2.11	5.17	1.292921	2.05E-11	yes	NAA15	N-alpha-acetyltransferase 15, NatA auxiliary subunit isoform X1
rna43608	0.001	2.61	11.34983	3.07E-05	yes	NAA38	N-alpha-acetyltransferase 38, NatC auxiliary subunit isoform X2
rna6655	0.001	0.68	9.409391	1.52E-08	yes	NAA40	N-alpha-acetyltransferase 40 isoform X1
rna11296	0.001	1.71	10.73978	1.93E-06	yes	SIRT3	NAD-dependent protein deacetylase sirtuin-3, mitochondrial isoform X2
